# ^99m^Tc activity concentrations in room air and resulting internal contamination of medical personnel during ventilation–perfusion lung scans

**DOI:** 10.1007/s00411-019-00793-2

**Published:** 2019-04-17

**Authors:** K. Brudecki, E. Borkowska, K. Gorzkiewicz, M. Kostkiewicz, T. Mróz

**Affiliations:** 1 Institute of Nuclear Physics, Polish Academy of Sciences, Radzikowskiego 152, 31-342 Kraków, Poland; 2 Electroradiology Department, Faculty of Health Sciences, Institute of Physiotherapy, Jagiellonian University, Collegium Medicum, Michalowskiego 12, 31-126 Kraków, Poland; 3 Heart and Vascular Diseases Department, Faculty of Medicine, Institute of Cardiology, Jagiellonian University, Collegium Medicum, Prądnicka 80, 31-202 Kraków, Poland; 4 Nuclear Medicine Department, John Paul II Hospital, Prądnicka 80, Kraków, Poland; 5 Institute of Physics, Jagiellonian University, Kraków, Poland

**Keywords:** ^99m^Tc, Air, Doses, Nuclear medicine, Medical personnel

## Abstract

This paper presents the results of measurements of ^99m^Tc activity concentrations in indoor air in a nuclear medicine department and resulting estimated ^99m^Tc intake by medical personnel. ^99m^Tc air activity measurements were conducted at the Nuclear Medicine Department, John Paul II Hospital, Krakow, Poland, during ventilation–perfusion SPECT lung scans. Technetium from the air was collected by means of a mobile aerosol sampler with a Petryanov filter operating at an average flow rate of 10 dm^3^ min^−1^. Measured activities ranged from 99 ± 11 to 6.1 ± 0.5 kBq m^−3^. The resulting daily average intake of ^99m^Tc by medical staff was estimated to be 5.4 kBq, 4.4 kBq, 3.0 kBq and 2.5 kBq, respectively, for male technicians, female technicians, male nurses and female nurses. Corresponding annual effective doses were 1.6 µSv for technicians and 1 µSv for nurses. The highest equivalent dose values were determined for extrathoracic (ET) airways: 5 µSv and 10 µSv for nurses and technicians, respectively. It is concluded that estimated annual absorbed doses are over three orders of magnitude lower than the dose limit established in the Polish Atomic Law Act and in recommendations of the International Commission on Radiological Protection for medical staff.

## Introduction

Technetium (Tc) is a chemical element belonging to the seventh group of the periodic table (transition metal). Primarily, Tc is obtained artificially by fission processes of heavy nuclei, but it is also present in the Earth’s crust due to spontaneous fission in uranium and thorium ores. There are 32 radioactive isotopes and 11 metastable isomers of technetium known at this moment.

One of the isotopes of technetium, namely ^99m^Tc, is frequently used in nuclear medicine, especially in diagnostics with the Single-Photon Emission Computed Tomography (SPECT) imaging technique. Physicochemical properties such as low energy of the gamma-rays emitted during decay (140.5 keV, emission probability *p* = 99%), short physical half-life of 6.01 h and rich co-ordination chemistry (which allows creation of many useful chemical complexes) are the reasons for the wide-spread use of ^99m^Tc in a wide range of diagnostic applications. It has been estimated that ^99m^Tc is the most frequently applied radioactive isotope for examinations in nuclear medicine (IAEA 2008).

A ventilation–perfusion lung scan is one of the medical imaging methods in which ^99m^Tc is applied. This examination consists of two lung scans. The first part, the perfusion scan, allows assessment of the lungs’ blood supply and requires intravenous injection of technetium macro aggregated albumin containing 150 MBq of ^99m^Tc. The second scan, the pulmonary ventilation scan, allows examination of the patency of the bronchial tree and lungs. Before scanning, a gas containing 400–500 MBq of ^99m^Tc is inhaled by the patient through a mouthpiece or mask covering the nose and mouth. In the medical routine, technetium DTPA (diethylenetriaminepentaacetic acid) or Technegas (ultrafine dispersion of ^99m^Tc-labeled carbon) is used as a source of the radionuclide (Jogi et al. 2010).

At the time or shortly after inhalation of gaseous radioactive technetium, part of the ^99m^Tc activity may leak into the room air and pose an additional risk of radiation burden for medical staff who perform examinations. To verify this hypothesis, a series of measurements of ^99m^Tc activity in the air were performed. The experiment was carried out in a nuclear medicine department, during routine ventilation–perfusion lung scans.

## Materials and methods

### Location

The present study was conducted at the Nuclear Medicine Department, John Paul II Hospital in Krakow, Poland. This unit is specialized in SPECT diagnostics and is supplied with a Siemens Symbia T16 SPECT/CT hybrid device. The following treatment protocols are routinely performed: myocardial perfusion imaging, bone scintigraphy, renoscintigraphy, and ventilation–perfusion lung scans. The medical staff consists of nine specialists (three technicians, four nurses and two physicians).

The department is also equipped with a Cyclomedica Technegas generator that is utilized in the routine inhalation procedure. The department usually performs ventilation–perfusion lung scans once per week on Tuesday. Four patients per day and about 200 patients per year are usually diagnosed in the department.

### Aerosol sampling and measurement

**Fig. 1:**
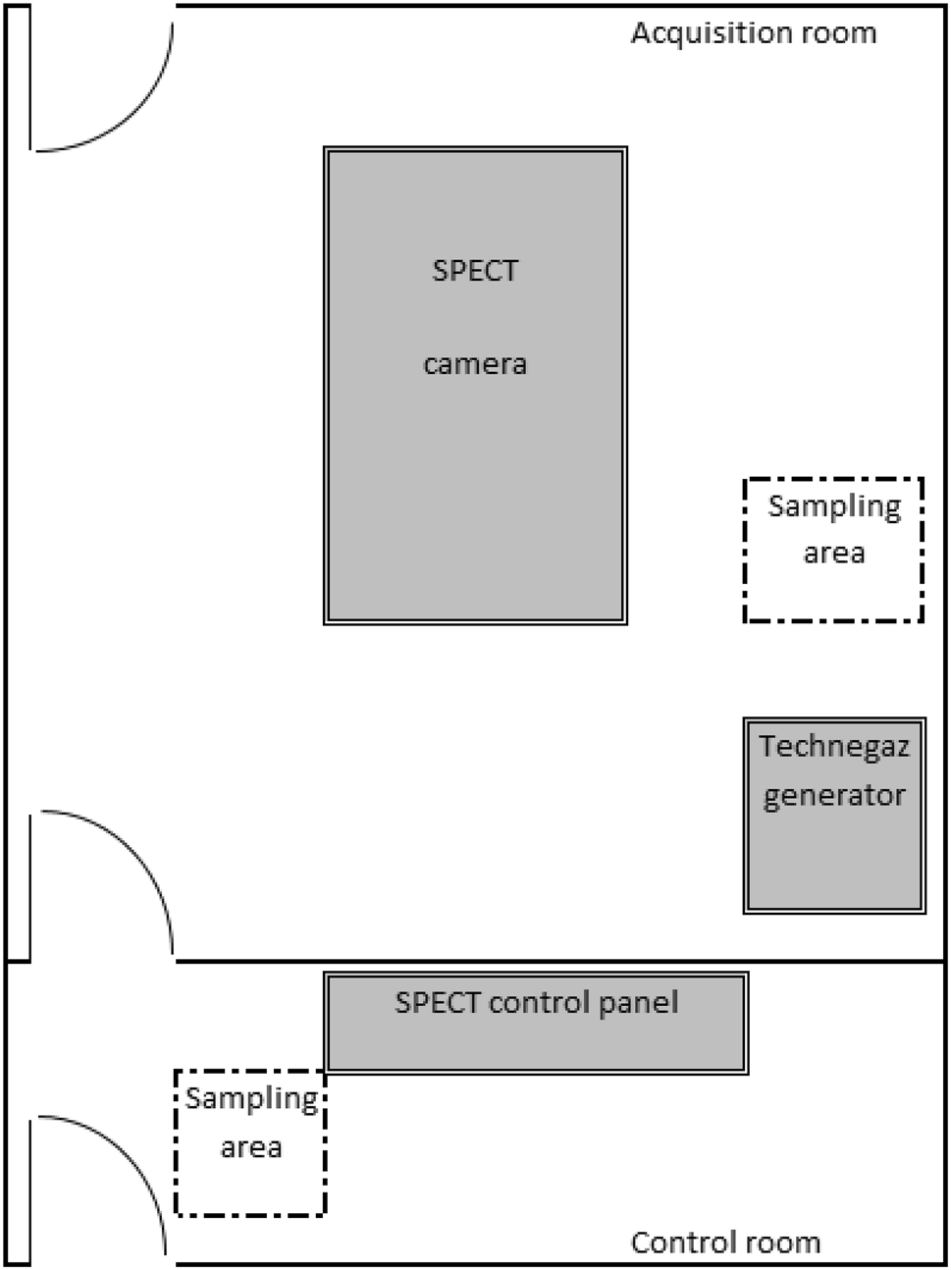
Layout of the rooms where air sampling was conducted

^99m^Tc activity concentration measurements were carried out during ventilation–perfusion lung scans on one specific day (10th September 2018). The experiment included collection of aerosol samples in two rooms—the first was a room with a SPECT camera and Technegas generator (called the acquisition room) and the second was a control room. The layout of the rooms is presented in Fig. 1. In each of the rooms, ten samples were gathered every 40 min to 1 h.

The rooms where the experiment was conducted were equipped with ventilation systems. Air exhaust rates were 1085 m^3^ h^−1^ and 60 m^3^ h^−1^, which gave ventilation efficiencies of about 8.5 and 2 air changes per hour for the acquisition and control room, respectively. During routine work, medical staff enters the acquisition room about twice per treatment, which results in additional ventilation of that room. The change of air volume due to door opening is difficult to estimate, but the authors believe that it is negligible compared with the highly efficient ventilation system.

Aerosols from the air were collected by means of a mobile aerosol sampler that operated at an average flow rate of 10 dm^3^ min^−1^. Aerosols were captured using a Petryanov filter FPP-15-1.5 (poly(vinyl chloride)) manufactured by ESFIL TEHNO AS (Estonia). Those filters are commonly used for aerosol collection, due to their high collection efficiency, which can reach up to 97% (Lipiński et al. 2013). Due to the short physical half-life of ^99m^Tc, right after sampling, filters were transported to the Institute of Nuclear Physics, Polish Academy of Science (Krakow, Poland). Subsequently, samples were compressed into pellets (5 cm diameter and about 4 mm height) and analyzed by means of a low-background gamma-ray spectrometer including a high-purity germanium (HPGe) detector manufactured by Ortec (GMX-30190-P, 30% relative efficiency). The electronics were based on NIM modules by Canberra and spectra acquisition and analysis were performed with Canberra Genie 2000 software. To determine ^99m^Tc activity collected on the Petryanov filter, the intensity of the 140 keV gamma-ray line was measured. Efficiency calibration was done with a multi-gamma source SZN 40/10 by Polatom. The source had a disc-shaped geometry of 50 mm diameter and 5 mm height. The matrix density was 1.00 g cm^−2^. Such method has already been successfully applied for measurements of aerosol and gas activities over the past years (Mietelski et al. 2005, 2014; Masson et al. 2011; Brudecki et al. 2018a).

### Dose estimation

Based on the measured ^99m^Tc activity concentrations in air, annual organ equivalent and effective doses for the technicians and nurses were estimated. In the beginning, utilizing routine working schemes, the average time spent by medical staff in contaminated areas was established. To perform one test, the technician entered the acquisition room twice and spent about 5 min there each time, once to assist the patient during inhalation of radioactive technetium and then to position him/her on the imaging table. After treatment, the technician entered the acquisition room a second time and helped the patient to get up and leave the diagnostic room. The technician spent the remaining time in the control room. To carry out a perfusion scan, the nurse needed to enter the acquisition room to perform an intravenous injection, which lasted approximately 5 min. Routinely, there are four ventilation–perfusion lung scans conducted in the department per day and week.

The next step involved estimation of the overall absorbed ^99m^Tc activities in the respiratory system and in other organs of the staff. Calculations were made using a biokinetic model of technetium recommended by the International Commission on Radiological Protection (ICRP 2016) in combination with the human respiratory tract model (HRTM) (ICRP 1994, 2002) and gastrointestinal tract model (GITM) (ICRP 1979), developed ICRP. Computer simulations were performed by means of the SAAM II software from the Epsilon Group. In line with ICRP recommendations, in the performed simulations, lung clearance of Technegas was assumed as a Type F (fast) (ICRP 2002). The Technegas system’s manufacturer ensures that the size of most of the produced aerosols is in the range of 30–60 nm (Cyclomedica 2007). Therefore, the deposition of ^99m^Tc aerosol fractions was calculated for an assumed activity median aerodynamic diameter (AMAD) of the attached aerosols of 50 nm. During work, the breathing rate of medical staff is adequate for light exercise. Aerosol deposition in HRTM for a 50 nm AMAD aerosol in light exercise is presented in Table 1. Other parameters used in the calculations are presented in Table 2.

**Table 1:** Aerosol deposition in the ICRP human respiratory tract model (HRTM) for a 50 nm activity median aerodynamic diameter (AMAD) and light exercise (*ET1* extrathoracic region, *ET2* posterior nasal passages, *BB* bronchial, *bb* bronchiolar, *AI* alveolar–interstitial) (ICRP 2002)

Region\exercise levels	Light exercise	
	♀ Female	♂ Male
ET1	3.2 × 10^−2^	3.2 × 10^−2^
ET2	3.4 × 10^−2^	3.4 × 10^−2^
BB fast and seq	5 × 10^−3^	4.7 × 10^−3^
BB slow	5 × 10^−3^	4.7 × 10^−3^
bb fast and seq	3.8 × 10^−2^	3.4 × 10^−2^
bb slow	3.8 × 10^−2^	3.4 × 10^−2^
AI	3.1 × 10^−1^	3.1 × 10^−1^

**Table 2:** Ventilation parameters and activity assumed in the calculations

Parameters\profession	Technician	Nurses
Exercise level	Light exercise	
Breathing rate (m^3^ h^−1^)	1.2 ♀, 1.5 ♂	
Daily intake (Bq)	4400 ♀, 5400 ♂	2450 ♀, 3000 ♂
Type of absorption	F (fast)	
Intake frequency	1 day a week (on Tuesday)	
Number of annual intakes	50	

♀ female, ♂ male

Finally, doses were calculated based on the time-integrated ^99m^Tc activity (model of technetium biokinetics and computer simulations), radiation-weighted coefficients (SECALL program, Oak Ridge National Laboratory, Oak Ridge, TN, USA) and tissue-weighting factors according to the ICRP methodology (ICRP 2007). More details on the applied methodology of absorbed dose calculations are given in Li et al. (2008) and Brudecki et al. (2014, 2017a, b, 2018a, b).

## Results and discussion

**Fig. 2:**
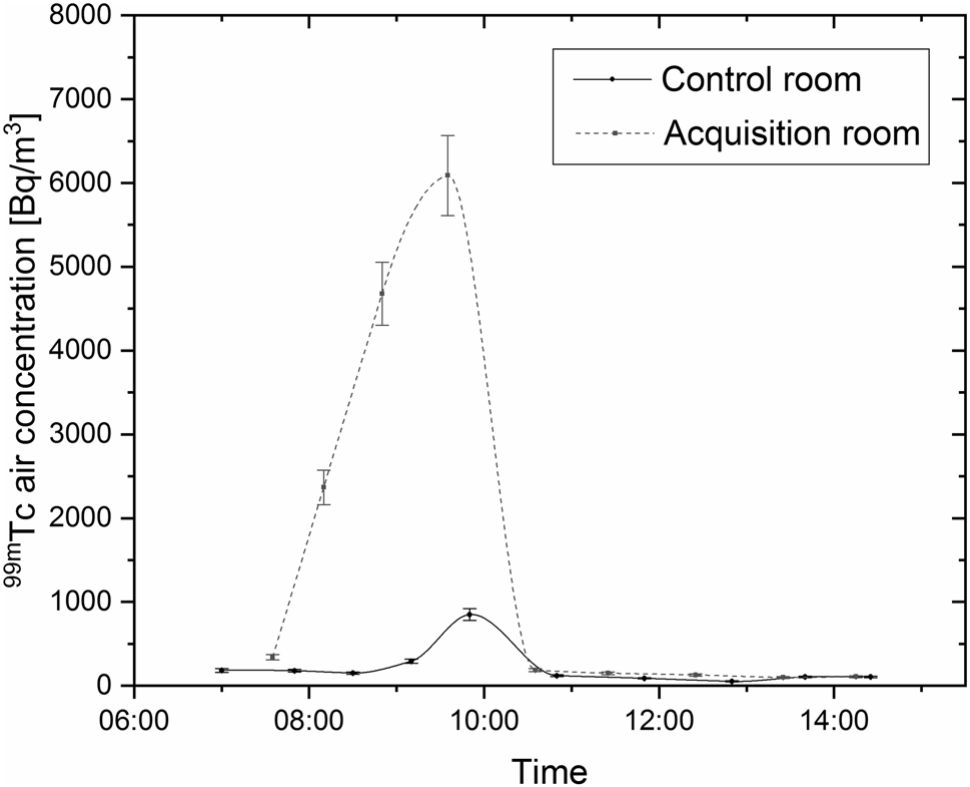
^99m^Tc air activity concentration in acquisition room and control room. The results are presented with one sigma uncertainty

At the time of the experiment, 20 aerosol samples were collected, measured and analyzed. In the acquisition room, ^99m^Tc activity concentration varied from 99 ± 11 Bq m^−3^ to 6.1 ± 0.5 kBq m^−3^. The activity of technetium systematically increased during the time the ventilation–perfusion lung scans were conducted. Soon after the last patient was examined, the concentration of ^99m^Tc rapidly declined as a result of air changing due to the highly effective ventilation system and frequent opening of doors. The activity of technetium in the control room reached 0.85 ± 0.07 kBq m^−3^, which is one order of magnitude lower than in the acquisition room. In addition, analysis of the samples collected before first utilization of the Technegas generator on the experiment day suggested that due to previous treatments conducted in the department, a constant radiation background from ^99m^Tc on the level of about 0.1–0.2 kBq m^−3^ existed (first samples collected in each room). The detailed results are presented in Fig. 2. It should be noted that, taking into consideration the sample collection times as well as the highly efficient ventilation systems installed in the department rooms, short-term ^99m^Tc activity concentrations may have significantly exceeded the determined values.

**Fig. 3:**
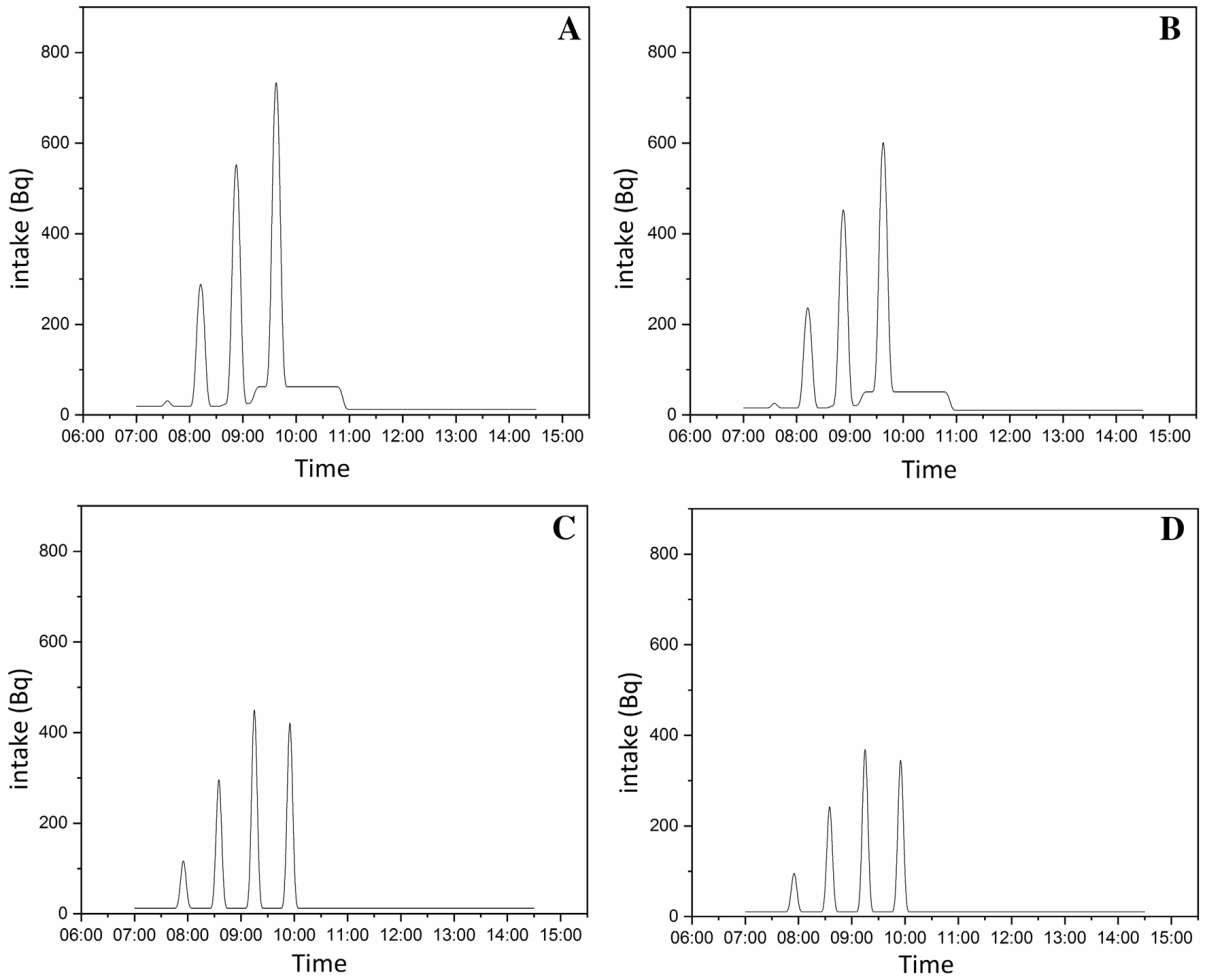
Estimation of daily ^99m^Tc intake: **a** male technician; **b** female technician; **c** male nurse; **d** female nurse. Area under curve: 5400 Bq, 4400 Bq, 3000 Bq and 2450 Bq, respectively, for male technician, female technician, male nurse and female nurse

On the basis of the outcome of the ^99m^Tc activity concentration measurements and taking into account the routine working time of technicians and nurses, daily activity intake was estimated. The results are as follows: 5.4 kBq, 4.4 kBq, 3.0 kBq and 2.5 kBq, respectively, for male technicians, female technicians, male nurses and female nurses. Detailed results are presented in Fig. 3.

The last step in the present study was the estimation of the dose received by the medical team involved. The annual effective committed dose reached 1.8 μSv for technicians and 0.9 μSv for nurses. The highest equivalent annual doses were found in ET airways—10 μSv, 9.5 μSv, 5.7 μSv and 5.3 μSv, respectively, for male technicians, female technicians, male nurses and female nurses. The fact that the dose for nurses (both sexes) was a factor of 2 lower than that for technicians is the result of a shorter time of work in the contaminated area, namely, in the acquisition and control rooms. Detailed information is presented in Table 3.

**Table 3:** Estimated annual organ equivalent and effective committed doses (given in Sv)

	Technician				Nurse			
	♀ Female		♂ Male		♀ Female		♂ Male	
	Daily	Annual	Daily	Annual	Daily	Annual	Daily	Annual
Adrenals	8.2 × 10^−9^	4.1 × 10^−7^	8.1 × 10^−9^	4.0 × 10^−7^	4.6 × 10^−9^	2.3 × 10^−7^	4.5 × 10^−9^	2.2 × 10^−7^
Bladder wall	3.4 × 10^−8^	1.7 × 10^−6^	3.2 × 10^−8^	1.6 × 10^−6^	1.9 × 10^−8^	9.4 × 10^−7^	1.8 × 10^−8^	8.9 × 10^−7^
Bone surfaces	1.8 × 10^−8^	8.8 × 10^−7^	1.7 × 10^−8^	8.6 × 10^−7^	9.8 × 10^−9^	4.9 × 10^−7^	9.6 × 10^−9^	4.8 × 10^−7^
Brain	2.6 × 10^−9^	1.3 × 10^−7^	2.7 × 10^−9^	1.4 × 10^−7^	1.4 × 10^−9^	7.1 × 10^−8^	1.5 × 10^−9^	7.6 × 10^−8^
Breasts	2.8 × 10^−9^	1.4 × 10^−7^	2.8 × 10^−9^	1.4 × 10^−7^	1.5 × 10^−9^	7.7 × 10^−8^	1.5 × 10^−9^	7.7 × 10^−8^
St wall	6.3 × 10^−8^	3.2 × 10^−6^	6.8 × 10^−8^	3.4 × 10^−6^	3.5 × 10^−8^	1.8 × 10^−6^	3.8 × 10^−8^	1.9 × 10^−6^
SI wall	6.2 × 10^−8^	3.1 × 10^−6^	6.5 × 10^−8^	3.3 × 10^−6^	3.4 × 10^−8^	1.7 × 10^−6^	3.6 × 10^−8^	1.8 × 10^−6^
ULI wall	1.5 × 10^−7^	7.3 × 10^−6^	1.6 × 10^−7^	8.0 × 10^−6^	8.1 × 10^−8^	4.1 × 10^−6^	8.9 × 10^−8^	4.4 × 10^−6^
LLI wall	9.9 × 10^−8^	4.9 × 10^−6^	1.1 × 10^−7^	5.6 × 10^−6^	5.5 × 10^−8^	2.8 × 10^−6^	6.2 × 10^−8^	3.1 × 10^−6^
Colon	1.3 × 10^−7^	6.3 × 10^−6^	1.4 × 10^−7^	7.0 × 10^−6^	7.0 × 10^−8^	3.5 × 10^−6^	7.7 × 10^−8^	3.9 × 10^−6^
Kidneys	1.7 × 10^−8^	8.3 × 10^−7^	1.8 × 10^−8^	8.8 × 10^−7^	9.3 × 10^−9^	4.6 × 10^−7^	9.7 × 10^−9^	4.9 × 10^−7^
Liver	1.6 × 10^−8^	8.1 × 10^−7^	1.6 × 10^−8^	7.9 × 10^−7^	9.0 × 10^−9^	4.5 × 10^−7^	8.8 × 10^−9^	4.4 × 10^−7^
Muscle	7.1 × 10^−9^	3.6 × 10^−7^	7.1 × 10^−9^	3.6 × 10^−7^	4.0 × 10^−9^	2.0 × 10^−7^	4.0 × 10^−9^	2.0 × 10^−7^
Ovaries	3.3 × 10^−8^	1.7 × 10^−6^	–	–	1.8 × 10^−8^	9.2 × 10^−7^	–	–
Pancreas	1.4 × 10^−8^	7.2 × 10^−7^	1.4 × 10^−8^	7.0 × 10^−7^	8.0 × 10^−9^	4.0 × 10^−7^	7.7 × 10^−9^	3.9 × 10^−7^
Red marrow	8.5 × 10^−9^	4.3 × 10^−7^	8.8 × 10^−9^	4.4 × 10^−7^	4.7 × 10^−9^	2.4 × 10^−7^	4.9 × 10^−9^	2.4 × 10^−7^
ET airways	1.9 × 10^−7^	9.5 × 10^−6^	2.0 × 10^−7^	1.0 × 10^−5^	1.1 × 10^−7^	5.3 × 10^−6^	1.1 × 10^−7^	5.7 × 10^−6^
Lungs	1.7 × 10^−8^	8.6 × 10^−7^	1.8 × 10^−8^	9.2 × 10^−7^	9.6 × 10^−9^	4.8 × 10^−7^	1.0 × 10^−8^	5.1 × 10^−7^
Skin	3.0 × 10^−9^	1.5 × 10^−7^	3.1 × 10^−9^	1.6 × 10^−7^	1.7 × 10^−9^	8.4 × 10^−8^	1.7 × 10^−9^	8.6 × 10^−8^
Spleen	9.6 × 10^−9^	4.8 × 10^−7^	9.8 × 10^−9^	4.9 × 10^−7^	5.3 × 10^−9^	2.7 × 10^−7^	5.4 × 10^−9^	2.7 × 10^−7^
Testes	–	–	4.1 × 10^−9^	2.1 × 10^−7^	–	–	2.3 × 10^−9^	1.1 × 10^−7^
Thymus	4.3 × 10^−9^	2.1 × 10^−7^	3.9 × 10^−9^	2.0 × 10^−7^	2.4 × 10^−9^	1.2 × 10^−7^	2.2 × 10^−9^	1.1 × 10^−7^
Thyroid	7.9 × 10^−8^	3.9 × 10^−6^	8.3 × 10^−8^	4.2 × 10^−6^	4.4 × 10^−8^	2.2 × 10^−6^	4.6 × 10^−8^	2.3 × 10^−6^
GB wall	2.3 × 10^−8^	1.2 × 10^−6^	2.2 × 10^−8^	1.1 × 10^−6^	1.3 × 10^−8^	6.4 × 10^−7^	1.2 × 10^−8^	6.1 × 10^−7^
HT wall	5.9 × 10^−9^	2.9 × 10^−7^	5.7 × 10^−9^	2.8 × 10^−7^	3.3 × 10^−9^	1.6 × 10^−7^	3.2 × 10^−9^	1.6 × 10^−7^
Uterus	2.2 × 10^−8^	1.1 × 10^−6^	2.1 × 10^−8^	1.1 × 10^−6^	1.2 × 10^−8^	6.1 × 10^−7^	1.2 × 10^−8^	5.9 × 10^−7^
Remainder	8.3 × 10^−9^	4.2 × 10^−7^	8.4 × 10^−9^	4.2 × 10^−7^	4.6 × 10^−9^	2.3 × 10^−7^	4.7 × 10^−9^	2.3 × 10^−7^
Effective dose	3.5 × 10^−8^	1.8 × 10^−6^	3.5 × 10^−8^	1.8 × 10^−6^	2.0 × 10^−8^	9.8 × 10^−7^	2.0 × 10^−8^	9.8 × 10^−7^

*St stomach* SI small intestine, *ULI* upper large intestine, *LLI* lower large intestine, *ET* extrathoracic, *GB* gallbladder, *HT* heart

Internal and external exposures of medical personnel due to ^99m^Tc have been described in some previous publications. Krajewska and Pachocki (2013) reported activities of ^99m^Tc in thyroids of examined medical staff in the range from 50 to 1800 Bq, with an average activity of 1500 Bq. Internal contamination was also studied by Ferdous et al. (2012) based on monitoring of ^99m^Tc activity in urine samples. Their reported values ranged from 9 to 314 Bq L^−1^ and corresponding doses varied from 0.4 to 15 μSv. Similar examinations were performed by Greaves et al. (1995) who collected 24 air samples during ventilation and imaging tests. They found an average ^99m^Tc activity concentration in air of 4.4 kBq m^−3^, which implied a daily effective dose to medical staff of 0.004 µSv. These authors reported also that 10 min after inhalation, ^99m^Tc activity concentrations in air decreased to background level (except for two samples where the concentration was less than 1.1 kBq m^−3^).

External dose rates reported by Smart (2004) for technicians working with ^99m^Tc were up to 100 μSv h^−1^. This value is similar to 43 μSv h^−1^ reported by Sattari et al. (2004). Another study (Forsa and Standen 2012) reported values of external radiation doses caused by ^99m^Tc during various medical procedures, with an average of 1 nSv per MBq assumed as a representative value for Norwegian nuclear medicine departments. Direct comparison of both external and internal doses suggests that external exposure is responsible for most of the effective annual dose.

## Conclusions

In the present study, ^99m^Tc activity concentrations in indoor air of a nuclear medicine hospital, as well as resulting absorbed doses for medical staff, were estimated. The study involved measurement and analysis of 20 aerosol samples collected in both the acquisition room and control room in the Nuclear Medicine Department, John Paul II Hospital, Krakow, Poland, during ventilation–perfusion lung scans. Although the technetium activity in the air and the estimated ^99m^Tc intakes seem to be quite high, corresponding annual effective doses are rather low. Furthermore, calculated annual effective doses are over three orders of magnitude lower than the dose limit established in the Polish Atomic Law Act and recommended by the ICRP (corresponding to effective doses from 6 to 20 mSv per year). In the light of current knowledge, such doses do not pose any significant health risk for medical staff. However, the present study concerned only a small part of the daily working routine of medical personnel and, therefore, does not necessarily imply that ^99m^Tc intake and associated doses will always be at sufficiently low and safe levels. Therefore, regular monitoring of internal contamination of hospital staff working with ^99m^Tc is recommended. Furthermore, it should be mentioned that conducting more measurements would be desirable; however, currently this is not feasible because the sample collection technique used in the present study is highly burdensome for both medical staff and patients. Due to this, the presented results must be interpreted with caution and should be seen just as a first indication of ^99m^Tc exposure in current clinical practice.
